# Cancellation of the zeroth order by a low-contrast grating

**DOI:** 10.1038/srep16501

**Published:** 2015-11-12

**Authors:** Bo Wang

**Affiliations:** 1 School of Physics and Optoelectronic Engineering, Guangdong University of Technology, Guangzhou 510006, China

## Abstract

The cancellation of the 0th order is described by a low-contrast fused-silica grating. In reported works, the high-contrast grating and complicated structure were applied with reasonable and excellent performance. However, the low-contrast grating is proved that it can also cancel the 0th order with the period more than 2λ in this paper. Grating parameters are optimized by using rigorous coupled-wave analysis, whose physical essence for cancellation of the 0th order can be well explained by modal method. The fabrication tolerance is investigated for production of the low-contrast grating for cancellation of the 0th order, which can be potentially used for writing the fiber Bragg grating.

Fiber Bragg gratings can be used for the optical differentiation^1^, the space division multiplexing^2^, and the picosecond optical signal processing^3^. It is an attractive prospect to investigate effective methods to fabricate fiber Bragg gratings, which have been reported by the two-beam holography^4^ and the zeroth-order nulled phase mask^5^. For the two-beam holography, a polymer/liquid crystal-based fiber Bragg grating was fabricated by photo-induced modulation, which could develop a new platform of integrated fiber systems^4^. For the zeroth-order nulled phase mask, the retroreflecting fiber Bragg grating was written in Ge-doped telecom fiber. Index modulations of 1.9 × 10^−3^ were exhibited after exposure with pulsed 800 nm femtosecond radiation^5^. Recently, subwavelength gratings with high spatial frequency^6^,^7^,^8^ have been proposed as novel micro-optical elements with advantages^9^,^10^,^11^ over conventional optical devices. It seems more interesting if a grating with high spatial frequency can be used to write a fiber Bragg grating directly^12^. Higher index contrast grating has been applied to extinguish the 0th order with excellent performance^13^,^14^. The cancellation of the 0th order is presented by a phase grating etched in Si_3_N_4_ layer on the substrate of fused silica. Although the zeroth-order nulled mask is exhibited, the grating structure is much complicated. In fact, standard fused-silica gratings can also extinguish the 0th order with period more than 2λ, which is different from high-contrast gratings^13^,^14^. To our knowledge, no one has presented such a zeroth-order nulled grating mask by the vector grating theory.

In this paper, a standard fused-silica grating is firstly proposed for cancellation of the 0th order with the vector grating theory. Such a low-contrast grating can work as a zeroth-order nulled mask to fabricate fiber Bragg grating for not only TE polarization but also TM polarization simultaneously. For possibility of cancellation of the 0th order, grating period and depth are optimized. To manufacture such a grating, fabrication tolerance for grating duty cycle and depth is investigated.

## Results

**Figure 1:**
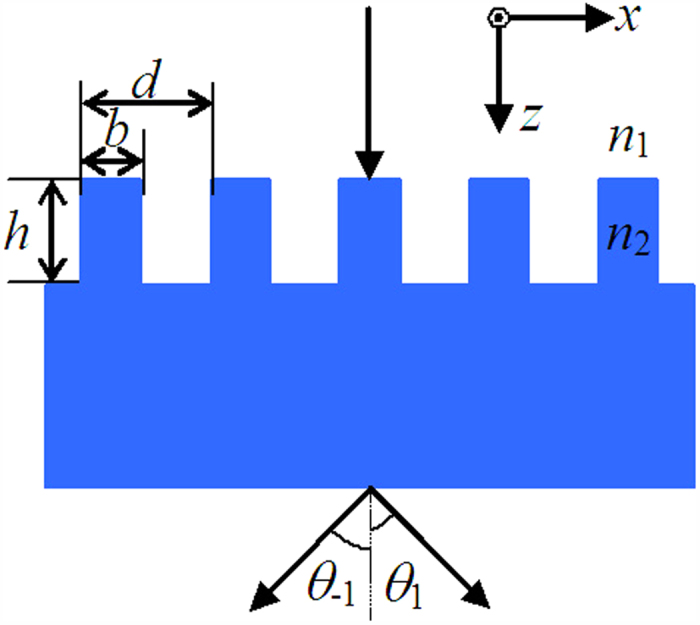
Schematic of zeroth-order nulled mask by a low-contrast grating (refractive indices *n*_1_: air, *n*_2_: fused silica; *d* period; *b* ridge width; *h* depth; *θ*_−1_ and *θ*_1_ diffraction angle of the −1st and the 1st order, respectively). The grating can be directly etched in substrate of fused silica.

The Schematic of a low-contrast grating for cancellation of the 0th order is shown in Fig. 1, where *d* is period, *b* is ridge width, and *h* is depth. The grating duty cycle *f* is defined as the ratio of the grating ridge width to the period. Such a standard grating is etched in fused silica with the refractive index *n*_2_ = 1.45. A plane wave illuminates the zeroth-order nulled grating under normal incidence with the incident angle *θ*_*i*_ = 0. The energy is mainly coupled into the −1st and the 1st orders, where the 0th order is extinguished. Such a standard grating can extinguish the 0th order with period more than 2λ after optimization by the vector grating theory.

**Figure 2:**
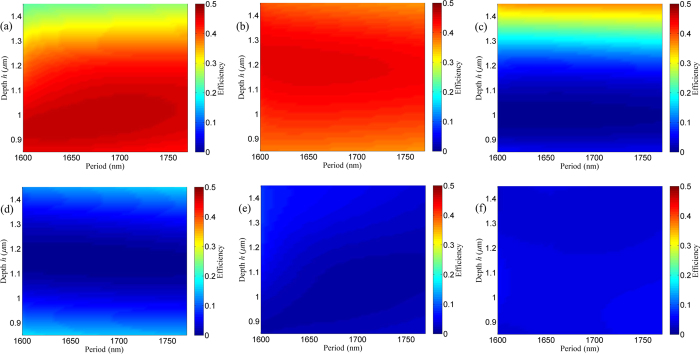
Efficiency for the low-contrast grating versus grating period and depth: (a) TE polarization in the 1st order, (b) TM polarization in the 1st order, (c) TE polarization in the 0th order, (d) TM polarization in the 0th order, (e) TE polarization in the 2nd order, and (f) TM polarization in the 2nd order. Different color corresponds to different efficiency from 0 to 50% as shown in the scaling of pseudocolor.

The main difference from the reported works^13^,^14^ is the low-contrast grating instead of the high-contrast grating related to cancellation of the 0th order by a standard grating. Hence, the first thing is to investigate the possibility by applying the novel period against the conventional limit of λ~2λ. For the diffraction analysis of such a high-spatial-frequency grating, the vector grating theory should be used such as the rigorous coupled-wave analysis (RCWA)^15^ and modal method^16^,^17^,^18^. Figure 2 shows efficiency of the 1st order for the low-contrast grating versus grating period and depth for both TE and TM polarizations at an incident wavelength of 800 nm with duty cycle of 0.3. In Fig. 2, with period of *d* = 1680 nm and *h* = 1.07 μm efficiencies of 46.15% and 43.60% can be diffracted into the 1st order for TE and TM polarizations, respectively. For the 0th order, efficiencies are no more than 1.3% for TE and TM polarizations. For the 2nd order, efficiencies are 1.16% for TE polarization and 4.29% for TM polarizations. References^13^,^14^ have reported reasonable and excellent performance about the reduction of 0th-order diffraction to the efficiency less than 0.1%. In this paper, as can be seen from Fig. 2(c,d), the efficiency less than 0.1% can be achieved within 1600 nm < *d* < 1615 nm and 0.99 μm < *h* < 1.01 μm for TE polarization or 1640 nm < *d* < 1685 nm and 1.14 μm < *h* < 1.17 μm for TM polarization. It can be seen that the 0th order is extinguished well by the low-contrast grating for both two polarizations simultaneously.

## Discussion

**Figure 3:**
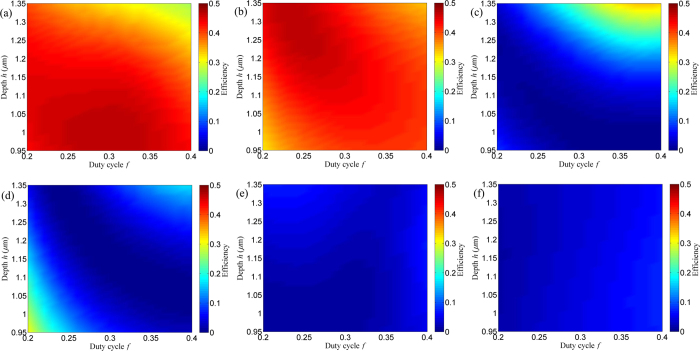
Efficiency of the 1st order versus grating duty cycle and depth with period of 1680 nm: (a) TE polarization in the 1st order, (b) TM polarization in the 1st order, (c) TE polarization in the 0th order, (d) TM polarization in the 0th order, (e) TE polarization in the 2nd order, and (f) TM polarization in the 2nd order. Different color corresponds to different efficiency from 0 to 50% as shown in the scaling of pseudocolor.

From the investigation especially for the period, one can see that the standard low-contrast grating can work as a zeroth-order nulled mask for the given special duty cycle of 0.3. However, due to less control of etching conditions, grating parameters, for example duty cycle and depth, can vary from optimized results. It is necessary to study the fabrication tolerance for manufacture in practice. Figure 3 shows efficiency of the 1st order versus grating duty cycle and depth. With deviations of duty cycle and depth from optimized results, the efficiency in the 1st order may be low and the efficiency in the 0th order may increase. However, with the fabrication tolerance range of 0.29 < *f* < 0.31 and 1.05 μm < *h* < 1.11 μm, efficiencies in the 1st order are more than 45.44% and 43.11% for TE and TM polarizations, respectively. In addition, efficiencies in the 0th order are less than 3% for both two polarizations.

It should be noted that there are some differences between the reference^14^ and the work in this paper. First, the high-contrast grating and complicated structure were applied in reference^14^. While in this paper, the low-contrast grating and simple structure are used to cancel the 0th order. Second, due to high-contrast grating in the reference^14^, reflections are important for the diffraction process. Therefore, the reference^14^ mainly studied multiple reflections in the modal method. In this paper, the reflection can be neglected for the low-contrast grating. Higher diffraction efficiency can be achieved compared with the reference^14^. Third, the 0th order nulled mask in the reference^14^ can work only for TE polarization. In this paper, the 0th order can be cancelled for both TE and TM polarizations simultaneously. In addition, the grating period is set to λ−2λ with the 0th and the ±1st orders in reference^14^. In this paper, the period more than 2λ is chosen to cancel the 0th order by the low-contrast grating. Although there may be five orders: the 0th, the ±1st and the ±2nd, the 0th can be cancelled and the ±2nd can be suppressed by the RCWA and modal method.

High-contrast gratings are usually composed of a high index contrast grating and a silica substrate. The high index layer needs to be deposited on the substrate. The fabrication process is complicated and less control in experiments will affect the performance. However, low-contrast gratings can be directly etched in the silica substrate. The grating structure is simple and the experimental condition has been optimized well by inductively coupled plasma technology. Therefore, low-contrast gratings can be potentially fabricated with low cost.

## Methods

As can be seen from given results, a low-contrast grating can also extinguish the 0th order, which is different from high-contrast gratings reported^13^,^14^. However, the physical essence can be established based on modal method. The incident wave will excite different modes in the grating region^19^, which meet the eigenfunction for TE polarization:


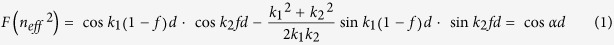


For TM polarization, the equation can be expressed as


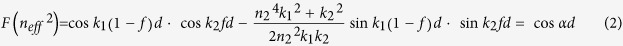


Where


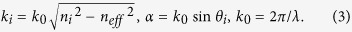


**Figure 4:**
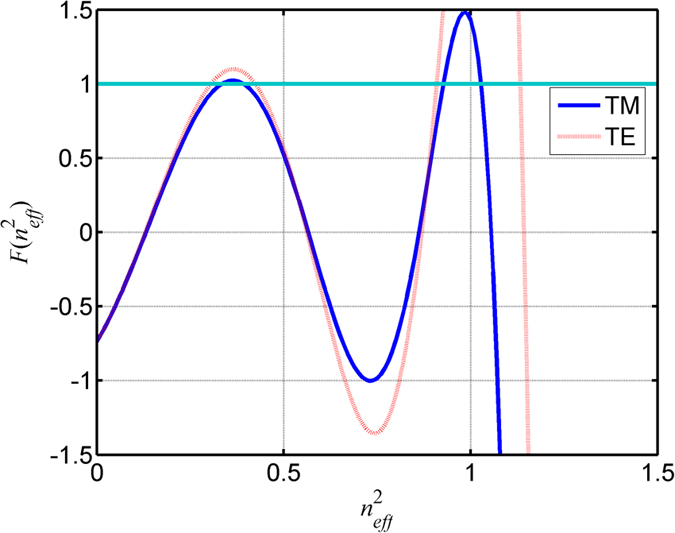
Eigenvalue-relation *F*(*n*_*eff*_^2^) with the duty cycle of 0.3 and period of 1680 nm. The intersections between *F*(*n*_*eff*_^2^) and −1 determine excited modes.

Figure 4 shows eigenvalue-relation *F*(*n*_*eff*_^2^) with the duty cycle of 0.3, period of 1680 nm and incident wavelength of 800 nm. The intersections between *F*(*n*_*eff*_^2^) and −1 determine excited modes with different effective indices. There are five modes with different effective indices for each polarization: 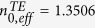, 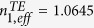, 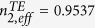, 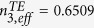, and 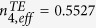 or 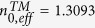, 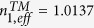, 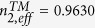, 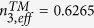, and 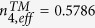.

**Figure 5:**
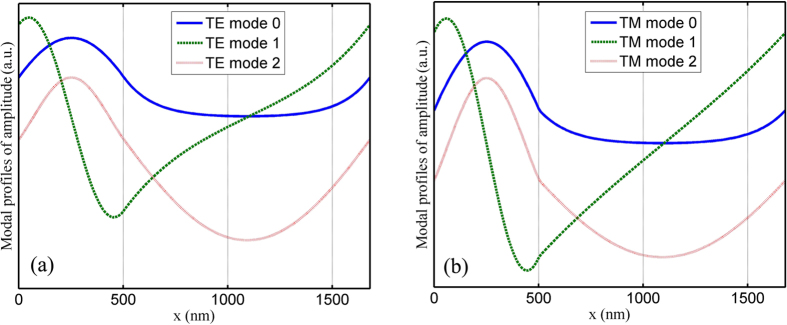
Mode profiles of amplitude of the first three propagating modes: (a) TE polarization and (b) TM polarization. Modes 0 and 2 are symmetrical, while mode 1 is asymmetrical.

Although five modes are excited by the incident wave, the excitation efficiencies are not the same for these modes. The energy exchange between the incident wave and excited modes is determined by the overlap integral^19^.


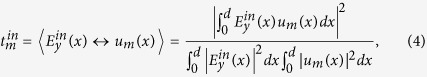


where *u*_*m*_(*x*) is the electric field of the *m*th grating mode and 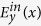 is that of the incident wave. By calculating the overlap integral, energy changes from the incident wave to modes 0 and 2 are 0.4726 and 0.5154 for TE polarization or 0.4217 and 0.4510 for TM polarization. Energy changes from the incident wave to modes 1, 3, and 4 are few, which can be omitted. Only modes 0 and 2 are considered during the simulation of diffracted orders. Figure 5 shows mode profiles of amplitude of the first three propagating modes. In Fig. 5, modes 0 and 2 are symmetrical, while mode 1 is asymmetrical.

Modes 0 and 2 are coupled into the diffracted orders with the phase difference


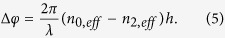


Efficiencies can be defined as^19^


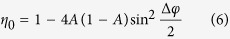



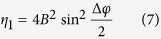


with A and B for transmission coefficients


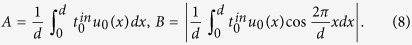


**Figure 6:**
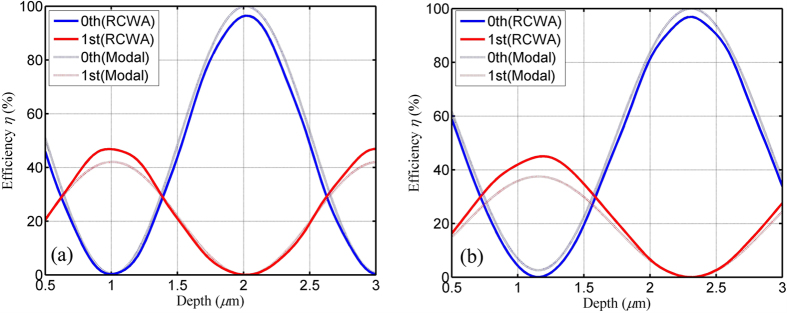
Comparison of efficiencies based on RCWA and modal method versus grating depth: (a) TE polarization and (b) TM polarization. The calculation by using RCWA coincides well with the simulation based on modal method.

In the next section, efficiencies in the 0th and the 1st orders will be discussed, respectively. On the one hand, in order to extinguish the 0th order, 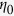 should be 0. With optimized grating parameters, A is 0.4692 for TE polarization and 0.4199 for TM polarization. And 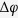 is 3.3352 for TE polarization and 2.9100 for TM polarization. By substituting A and 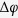 into Eq. (6), efficiencies in the 0th order are 1.31% and 3.87% for TE and TM polarizations, respectively. Therefore, the 0th order can be nearly cancelled by a low-contrast grating based on modal method. On the other hand, efficiencies in the 1st order can also be simulated by Eq. (7). For the optimized low-contrast grating, B is 0.3243 for TE polarization and 0.3061 for TM polarization. Figure 6 shows comparison of efficiencies based on RCWA and modal method versus grating depth for both TE and TM polarizations. One can see that efficiencies numerically calculated by using RCWA coincide well with predictions based on modal method. Therefore, the physical mechanism can be well given by the coupling of modes 0 and 2 for the cancellation of the 0th order by a low-contrast grating.

The cancellation of the 0th order has been shown by a low-contrast grating. For conventional cancellation of the 0th order, the grating period should be limited in the range of λ~2λ. However, a transmission grating has been proved to be impossible to extinguish the 0th order with the given limit. While applying the novel period more than 2λ against the conventional limit, the feasibility is exhibited for cancellation of the 0th order. Accurate grating parameters can be optimized by using RCWA. Modal method can give physical mechanism to the zeroth-order nulled grating mask. The fabrication tolerance is much moderate during manufacture. Although the period of a phase mask for fabrication of fiber Bragg gratings is fixed by the characterization of the fiber Bragg grating, the low-contrast grating provides the feasibility of cancellation of the zeroth order by changing the grating depth and duty cycle with the fixed period. Since the low-contrast grating has been optimized and demonstrate in experiments^19^, such a zeroth-order nulled grating mask can be potentially tested and demonstrated in experiments, which can be well used in the writing fiber Bragg grating.

## Additional Information

**How to cite this article**: Wang, B. Cancellation of the zeroth order by a low-contrast grating. *Sci. Rep.* **5**, 16501; doi: 10.1038/srep16501 (2015).
